# Reframing diagnostic reasoning: the Bayesian imperative in shoulder examination

**DOI:** 10.3389/fsurg.2026.1813508

**Published:** 2026-07-08

**Authors:** Eugene Rezk

**Affiliations:** 1 Department of Orthopedics and Traumatology, Military Hospital Vienna, Vienna, Austria; 2 Virtual and Augmented Reality Group, Institute for Visual Computing and Human-Centered Technology, Faculty of Informatics, TU Wien, Vienna, Austria; 3 Trauma Center Vienna—Meidling Site, Sigmund Freud Private University, Vienna, Austria; 4 Department of Physics and Biophysics, Medical University Graz, Graz, Austria; 5 Entrepreneurship in Digital Health (EDITH), Medical University of Graz, Graz, Austria

**Keywords:** Bayesian clinical reasoning, diagnostic accuracy, evidence-based clinical decision-making, likelihood ratios, pre-test/post-test probability, musculoskeletal diagnosis, sequential diagnostic testing, shoulder special tests

## Abstract

Despite the widespread use of clinical shoulder tests such as the Jobe or Neer test, diagnostic reasoning often remains binary and insufficiently quantitative. Moreover, reported diagnostic accuracy varies substantially across studies, with sensitivity, specificity, and likelihood ratios typically presented as ranges that do not adequately reflect study weighting or uncertainty. In this Perspective, we apply a Bayesian framework to formalize diagnostic reasoning by integrating pre-test probability with pooled likelihood ratios, using published data from Hegedus et al. (2012). This approach allows for the calculation of post-test probability in a transparent and reproducible manner. Using a clinical example, a pre-test probability of 30% increased to approximately 51% following a positive Drop Arm Test, illustrating that even tests with comparatively higher pooled likelihood ratios produce only moderate shifts in diagnostic probability. However, when multiple tests are applied sequentially, the combined effect results in a substantial increase in post-test probability (up to approximately 63%). Importantly, while the final post-test probability remains invariant to the order of test application due to the multiplicative nature of likelihood ratios, the intermediate diagnostic trajectory differs depending on the sequence, with implications for clinical decision-making and efficiency. These findings highlight the limited standalone diagnostic value of individual clinical tests and emphasize the importance of structured, sequential test application. Rather than relying on heterogeneous range-based summaries, Bayesian modeling provides a coherent and evidence-based framework for clinical reasoning that explicitly accounts for diagnostic uncertainty. As such, it offers a more robust approach for interpreting clinical test results in musculoskeletal care.

## 1 Introduction

The human shoulder is a highly mobile yet biomechanically complex joint, rendering it susceptible to both acute trauma and degenerative changes (1, 2). Shoulder disorders rank among the most common musculoskeletal complaints encountered in primary and orthopedic care. However, accurate diagnosis remains challenging due to the non-specific and overlapping nature of clinical symptoms, such as pain, reduced range of motion, and functional limitations, none of which are pathognomonic (3). To aid in clinical decision-making, physical examination maneuvers—such as the Jobe, Hawkins-Kennedy, and Neer tests—are frequently employed as first-line diagnostic tools.These tests aim to identify structural pathology such as rotator cuff tears, tendinopathies, and impingement syndromes (1, 3–7). However, the interpretation of these maneuvers tends to be dichotomous: positive or negative. This overly simplistic strategy isn’t congruent with the probabilistic nature of diagnostic reasoning, where results would ideally change the estimated probability of disease and not merely confirm or exclude it (8). Despite the decades of emphasis on evidence-based practice, use of quantitative diagnostic aids such as likelihood ratios and Bayesian post-test probability remains the exception in the day-to-day shoulder examination (16). Most decisions concerning advanced imaging—in particular, MRI—are made without explicit consideration of pre-test probability or diagnostic thresholds (9). Not only does this gap in diagnosis increase the risk of imaging overuse, but it also delays timely treatment of patients with a high likelihood of pathology. Lacking in clinical paradigms of today is a methodical, evidence-based approach to transforming physical examination data into useful diagnostic probabilities. Such an approach would aid decision-making by enabling clinicians to determine when conservative management is sufficient and when imaging or consultation by surgery is needed. Herein, we recommend reconsideration of shoulder test interpretation within Bayesian framework. Utilizing published test parameters of the tests, we approximate post-test odds under various scenarios of clinical findings and recommend threshold-based approach in directing subsequent downstream clinical actions. We would like to prompt for a more reasonable, patient-oriented, and evidence-based methodology towards shoulder diagnostics.

## 2 Methodology

We conducted a targeted, narrative review of published clinical studies evaluating the diagnostic accuracy of shoulder examination maneuvers. We aimed to extract relevant data in terms of sensitivity, specificity, and positive and negative likelihood ratios for tests commonly used, including the Jobe (empty can), Neer, Hawkins-Kennedy, Drop Arm, and Lift-Off tests. Due to substantial heterogeneity across included studies in terms of design, population, and reference standards, simple arithmetic pooling of sensitivity and specificity was not performed. Instead, diagnostic estimates were interpreted qualitatively, prioritizing findings from higher-level evidence sources, particularly systematic reviews and meta-analyses where available [e.g., (4)]. This approach avoids methodological bias introduced by inappropriate averaging across heterogeneous datasets. Our evidence base was systematic reviews and clinical literature by (1, 3–7) and other key references that contrasted test reproducibility, biomechanical specificity, and concordance with imaging-validated diagnoses (2, 10, 11). To further account for heterogeneity, we evaluated key methodological factors such as study design (prospective vs. retrospective), clinical setting (primary vs. tertiary care), population characteristics (age, symptom duration, activity level), and reference standards (MRI, arthroscopy, or clinical follow-up). These factors were considered during interpretation of the diagnostic metrics to avoid overgeneralization. Based on the derived data, Bayesian principles were used to model changes in post-test probability across a range of pre-test estimates. This allowed us to model clinical diagnostic shifts in practice and propose a more nuanced decision-making approach. Manual calculations were based on published sensitivity and specificity values, with results expressed as percentage probabilities to enhance clinical interpretability. Likelihood ratios were derived from reported sensitivity and specificity values at the individual study level. Given the heterogeneity of the included studies, these values should be interpreted as ranges rather than precise pooled estimates. Consequently, the reported likelihood ratios serve as approximate indicators of diagnostic performance rather than definitive summary measures.

## 3 Clinical tests and their analysis

### 3.1 Diagnostic accuracy of shoulder tests

**Figure 1:**
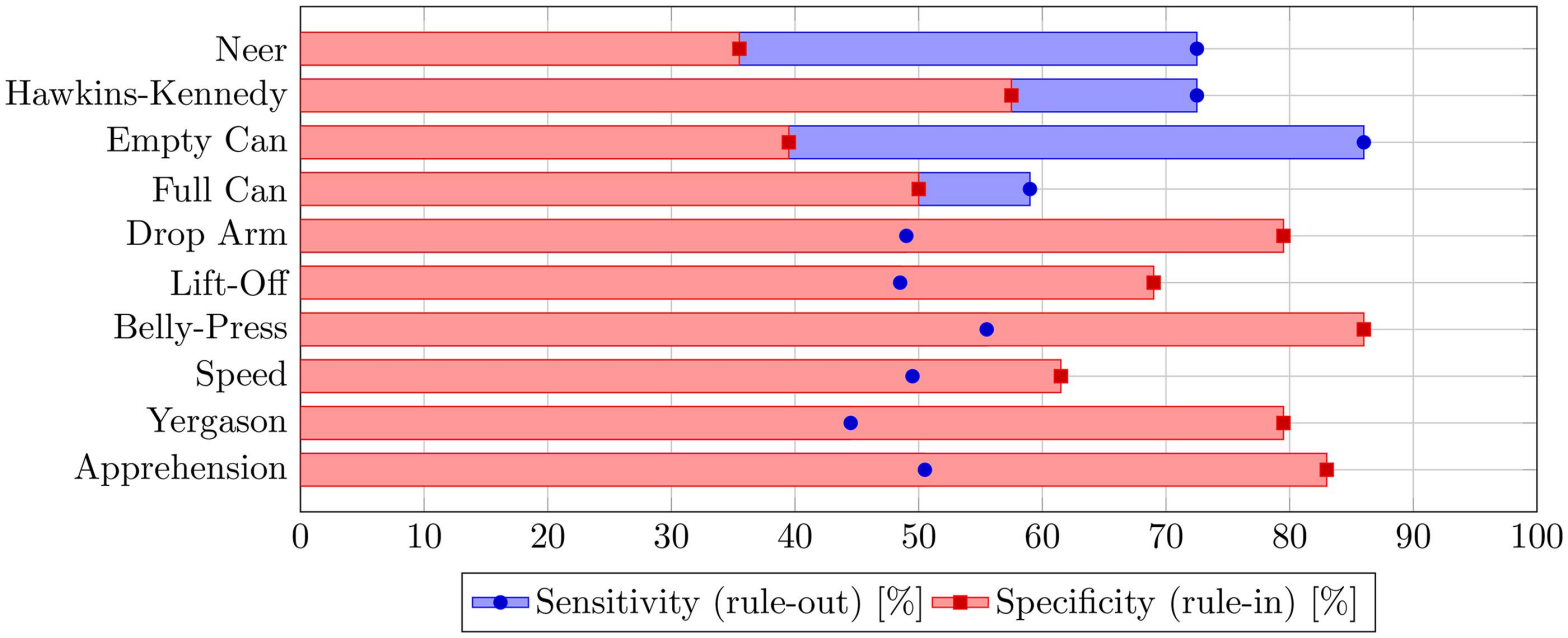
Diagnostic performance landscape of clinical shoulder tests based on Hegedus (4). Blue represents rule-out performance (sensitivity), red represents rule-in performance (specificity).

Diagnostic accuracy of clinical shoulder tests was assessed using sensitivity and specificity values extracted from five primary studies. Subacromial impingement tests (Neer, Hawkins-Kennedy) generally demonstrate higher sensitivity, supporting their role as screening tools to rule out pathology (1, 4, 7). Rotator cuff-specific tests (Drop Arm, Lift-Off, Belly-Press) show higher specificity and are therefore more useful for ruling in structural tendon pathology (5, 6). Biceps-related tests (Speed, Yergason) demonstrate moderate and inconsistent diagnostic performance and should be interpreted in combination with other clinical findings. The Apprehension Test shows moderate diagnostic accuracy for anterior instability (15). Given the heterogeneity in reported diagnostic accuracy across studies, the subsequent Bayesian modeling and case-based calculations were primarily based on pooled estimates reported by (4), which were considered the most methodologically robust and internally consistent dataset for the present analysis. This approach ensures coherence between the quantitative example and the underlying evidence base, as illustrated in Figure 1.

### 3.2 Positive and negative predictive value (PPV/NPV)

Positive and Negative Predictive Values describe the probability that a test result correctly reflects disease status:

$$\mathrm{PPV}=\frac{\mathrm{TP}}{\mathrm{TP}+\mathrm{FP}},\;\mathrm{NPV}=\frac{\mathrm{TN}}{\mathrm{TN}+\mathrm{FN}}$$

Both measures depend strongly on disease prevalence and therefore vary across clinical settings. Consequently, PPV and NPV are not suitable for cross-study comparison. For this reason, likelihood ratios were used as the primary metric for diagnostic evaluation.

## 4 Interpretation and application

### 4.1 Diagnostic reasoning framework

Diagnostic performance in shoulder examination is best described using sensitivity, specificity, and likelihood ratios (12). Sensitivity and specificity are intrinsic test properties, whereas likelihood ratios (${\mathrm{LR}}^{+}$ and ${\mathrm{LR}}^{-}$) allow prevalence-independent Bayesian updating of disease probability.

$${\mathrm{LR}}^{+}=\frac{\text{Sensitivity}}{1-\text{Specificity}},\;{\mathrm{LR}}^{-}=\frac{1-\text{Sensitivity}}{\text{Specificity}}$$

These measures form the basis for clinical decision-making by converting pre-test into post-test probabilities.

To address methodological concerns regarding the aggregation of diagnostic accuracy measures, pooled sensitivity and specificity values were adopted from the meta-analysis by Hegedus et al. (4), where available. These pooled estimates provide more robust and methodologically appropriate summary measures compared to simple arithmetic means across heterogeneous studies (1–7, 10, 11). To facilitate the clinical interpretation of these aggregated diagnostic accuracy measures, the pooled sensitivity and specificity values were further applied within a Bayesian framework. This approach enables the translation of summary test characteristics into clinically meaningful probability estimates by modelling their impact on diagnostic decision-making in an individual patient context. The following hypothetical example demonstrates how these pooled estimates can be used to update a predefined pre-test probability to a post-test probability of the underlying pathology.

## 5 Clinical application: Bayesian example

### 5.1 Case-based Bayesian diagnostic reasoning

Consider a 55-year-old patient presenting with progressive shoulder pain following a fall onto the outstretched arm during a low-energy household accident. The patient reports pain during overhead activities, night pain, and weakness when lifting the arm, which are typical clinical features suggestive of a rotator cuff lesion, particularly involving the supraspinatus tendon. Based on the clinical presentation, a pre-test probability of 30% for a supraspinatus tear is assumed. The corresponding pre-test odds are:

$$\text{Pre-test odds}=\frac{0.3}{1-0.3}=0.429$$

Likelihood ratios (LRs) were derived from sensitivity and specificity values reported by Hegedus et al. and applied sequentially to simulate a clinically realistic diagnostic pathway.

### 5.2 Sequential testing strategy (clinically oriented)

**Step 1: Empty Can Test (${\mathrm{LR}}^{+}\approx 1.42$)**

$$0.429\times 1.42=0.609\;\Rightarrow \;P=\frac{0.609}{1+0.609}\approx 0.38$$

**Step 2: Full Can Test (${\mathrm{LR}}^{+}\approx 1.18$)**

$$0.609\times 1.18=0.718\;\Rightarrow \;P=\frac{0.718}{1+0.718}\approx 0.42$$

**Step 3: Drop Arm Test (${\mathrm{LR}}^{+}\approx 2.39$)**

$$0.718\times 2.39=1.72\;\Rightarrow \;P=\frac{1.72}{1+1.72}\approx 0.63$$

This sequential approach increases the post-test probability from 30% to approximately 63%, representing a meaningful diagnostic gain with a balanced sensitivity–specificity trade-off.

### 5.3 Alternative sequence (reverse order)

To illustrate the effect of test ordering, the same likelihood ratios were applied in reverse sequence:

**Step 1: Drop Arm Test**

$$0.429\times 2.39=1.025\;\Rightarrow \;P=\frac{1.025}{1+1.025}\approx 0.51$$

**Step 2: Full Can Test**

$$1.025\times 1.18=1.21\;\Rightarrow \;P=\frac{1.21}{1+1.21}\approx 0.55$$

**Step 3: Empty Can Test**

$$1.21\times 1.42=1.72\;\Rightarrow \;P=\frac{1.72}{1+1.72}\approx 0.63$$

**Figure 2:**
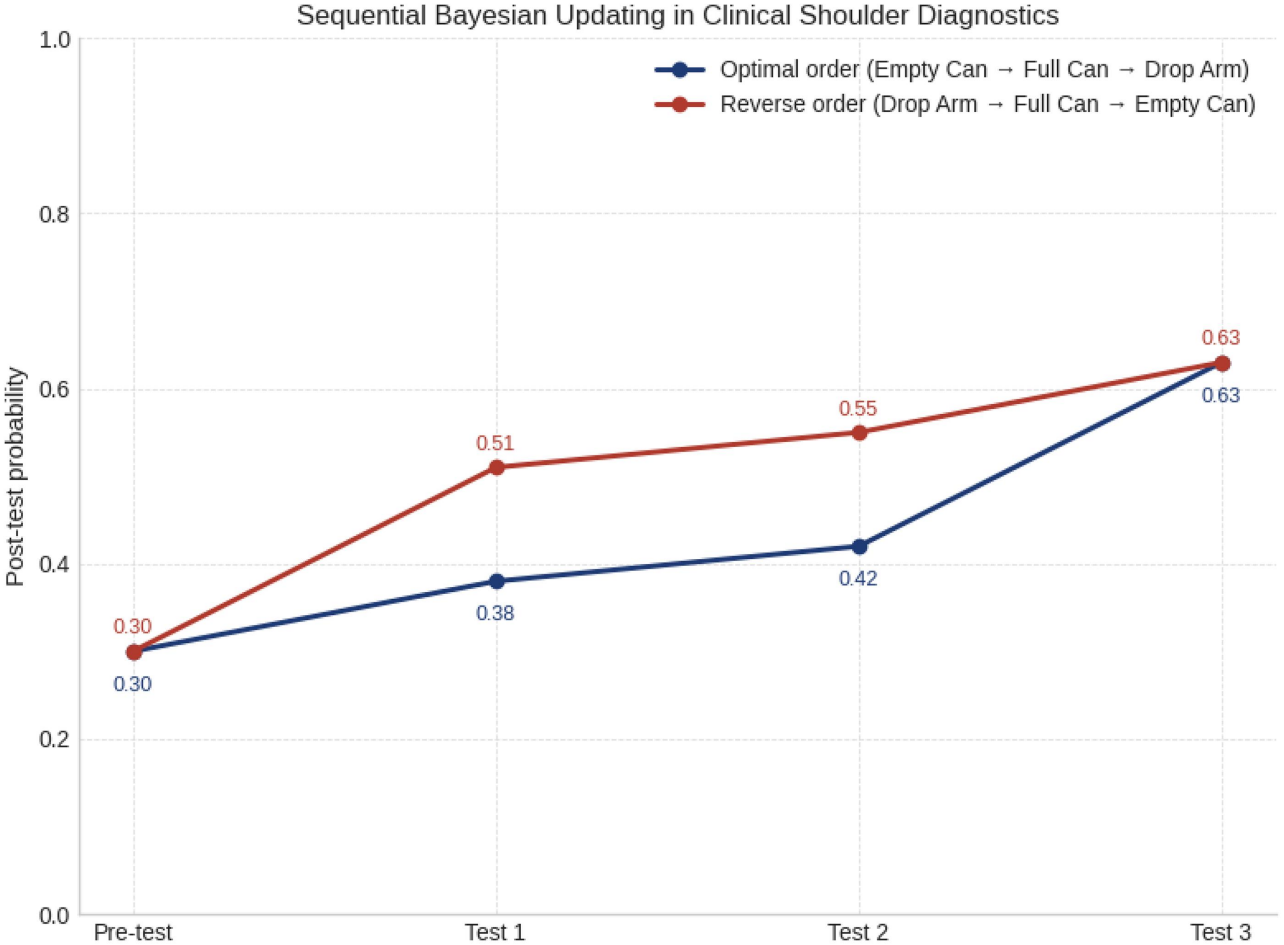
Graphical analysis of Bayesian diagnostic reasoning. **(Blue)** Clinically optimal testing sequence. **(Red)** Reverse sequence illustrating an alternative testing order.

This reverse sequence leads to the same final post-test probability but demonstrates how test ordering influences the intermediate diagnostic confidence and clinical decision thresholds. Notably, the final post-test probability remains identical (63%), reflecting the multiplicative and commutative nature of likelihood ratios see Figure 2.

## 6 Discussion

The present analysis demonstrates that clinical tests for supraspinatus tendon lesions exhibit substantial variability in diagnostic performance. Based on the calculated likelihood ratios, most tests show only limited diagnostic utility, with LR values close to 1, indicating minimal impact on post-test probability. Among the evaluated tests, the Drop Arm Test and Belly-Press Test demonstrated comparatively higher likelihood ratios, suggesting a greater role in ruling in pathology. However, even these tests did not consistently reach thresholds indicative of strong diagnostic performance. In our illustrative example, the application of a positive Drop Arm test result increased the post-test probability to approximately 51%. Conversely, a negative test would only modestly decrease the post-test probability, indicating that a lesion cannot be reliably excluded and that residual diagnostic uncertainty warrants consideration of alternative diagnoses or further assessment. Despite this increase, a relevant degree of diagnostic uncertainty remains, emphasizing that clinical tests should be interpreted within the broader clinical context rather than in isolation. These findings are consistent with previous literature, which has highlighted the limited standalone diagnostic value of individual shoulder examination maneuvers. Therefore the here introduced example demonstrates that individual clinical tests provide limited diagnostic value when used in isolation. However, their sequential combination results in a substantial increase in post-test probability. While Bayesian updating is mathematically order-independent, the clinical utility of diagnostic testing is strongly sequence-dependent, emphasizing the importance of structured and evidence-based test selection in musculoskeletal assessment. Although the final probability is mathematically independent of test order, the diagnostic pathway differs substantially. In the clinically oriented sequence, early application of sensitive tests facilitates reduction of diagnostic uncertainty, while subsequent use of more specific tests strengthens diagnostic confidence. In contrast, initiating the diagnostic process with a more specific test (e.g., Drop Arm) provides an early increase in probability but offers limited ability to exclude the condition if negative, thereby reducing clinical efficiency. To further explore the impact of diagnostic uncertainty, sensitivity analyses across different pre-test probabilities can be considered. Our scenario highlights that the diagnostic impact of a given test result is highly dependent on the initial clinical estimate of disease probability. Consequently, identical test results may lead to substantially different clinical conclusions depending on the context. The variability in sensitivity and specificity across studies further underscores the heterogeneity of clinical testing and patient populations. A key limitation of the present analysis is the reliance on aggregated sensitivity and specificity values rather than individual patient data or uniform study conditions. Additionally, variability in study design, sample size, and reference standards may influence the observed diagnostic performance (13–15). From a clinical perspective, these results support a combined and probabilistic approach to diagnosis rather than reliance on single physical examination tests. Likelihood ratios, when applied within a Bayesian framework, allow for more nuanced clinical decision-making and improved diagnostic reasoning by explicitly incorporating uncertainty. Future research should focus on standardizing test protocols and evaluating combinations of clinical tests in sequential diagnostic strategies to improve overall diagnostic performance in shoulder pathology. Furthermore, formal Bayesian models incorporating continuous ranges of pre-test probabilities may enhance the applicability of these findings in real-world clinical settings (17).

## 7 Conclusion

Clinical tests for supraspinatus tendon lesions demonstrate limited standalone diagnostic value, as both positive and negative results only moderately influence post-test probability. A probabilistic, Bayesian approach that integrates multiple clinical findings allows for a more nuanced and individualized estimation of diagnostic likelihood. Consequently, clinical tests should not be used in isolation but rather as part of a comprehensive diagnostic strategy, potentially complemented by imaging or further clinical assessment.

## Data Availability

The raw data supporting the conclusions of this article will be made available by the authors, without undue reservation.
